# Black carbon absorption at the global scale is affected by particle-scale diversity in composition

**DOI:** 10.1038/ncomms12361

**Published:** 2016-09-01

**Authors:** Laura Fierce, Tami C. Bond, Susanne E. Bauer, Francisco Mena, Nicole Riemer

**Affiliations:** 1 Department of Environmental and Climate Sciences , Brookhaven National Laboratory, Upton, New York 11973, USA; 2 Visiting Scientists Program, University Corporation for Atmospheric Research, Boulder, Colorado 80307, USA; 3 Department of Civil and Environmental Engineering, University of Illinois at Urbana-Champaign, Urbana, Illinois 61801, USA; 4 NASA Goddard Institute for Space Studies, New York City, New York 10025, USA; 5 The Earth Institute, Columbia University, New York City, New York 10025, USA; 6 Department of Atmospheric Sciences, University of Illinois at Urbana-Champaign, Urbana, Illinois 61801, USA

## Abstract

Atmospheric black carbon (BC) exerts a strong, but uncertain, warming effect on the climate. BC that is coated with non-absorbing material absorbs more strongly than the same amount of BC in an uncoated particle, but the magnitude of this absorption enhancement (*E*_abs_) is not well constrained. Modelling studies and laboratory measurements have found stronger absorption enhancement than has been observed in the atmosphere. Here, using a particle-resolved aerosol model to simulate diverse BC populations, we show that absorption is overestimated by as much as a factor of two if diversity is neglected and population-averaged composition is assumed across all BC-containing particles. If, instead, composition diversity is resolved, we find *E*_abs_=1−1.5 at low relative humidity, consistent with ambient observations. This study offers not only an explanation for the discrepancy between modelled and observed absorption enhancement, but also demonstrates how particle-scale simulations can be used to develop relationships for global-scale models.

## Abstract (web summary)

Model and laboratory experiments disagree with observations regarding the absorption properties of black carbon particles. Here, using a particle-resolved aerosol model, the authors show that when composition diversity is considered, absorption enhancement is consistent with ambient observations.

## Graphical abstract

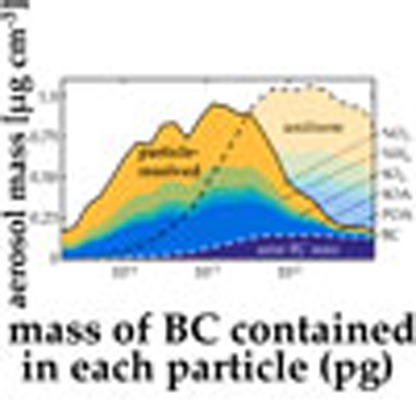

Black carbon (BC) is the most strongly absorbing component of atmospheric aerosol^1^, leading to strong atmospheric warming. Simulations of the global atmosphere are used to estimate this warming. One challenge in predicting absorption per BC mass is accounting for the particle microphysical details that affect light absorption and radiative transfer within these large-scale models. Within individual particles, BC is mixed with other aerosol components to varying degree^2^,^3^,^4^, depending on the emission source^5^,^6^,^7^,^8^ and the extent of atmospheric processing^9^,^10^,^11^,^12^,^13^. Modelled absorption by BC is strongly enhanced if BC is coated with non-absorbing aerosol components (for example, refs 14, 15), but ambient observations show weaker levels of absorption enhancement^15^,^16^,^17^. To our knowledge, refs 15, 16, 17 are the only studies that compare modelled and observed light absorption without significant measurement artifacts.

This discrepancy in modelled and observed light absorption indicates that the characteristics of BC-containing particles assumed in many models do not adequately represent BC-containing particles found in the atmosphere. Treatments of particle composition vary between global models, from simple mass-based schemes^18^,^19^, which do not track any information about particle size or composition, to modal^20^,^21^,^22^ and sectional^23^,^24^ schemes, which simulate the evolution of the particle size distribution but assume that particles within the same mode or of the same size have the same composition. Variants of these broad categories refine aspects of the particle population. For example, some sectional schemes represent separate particle sub-populations and track size-resolved composition within each population (for example, ref. 25). Because even the most sophisticated global models are not able to track characteristics of individual particles^22^,^25^, it has not been possible to explore how microscale particle details influence macroscale radiative effects.

This study investigates errors in modelled light absorption caused by ignoring diversity in particle composition, and shows that absorption is strongly affected by the distribution of components among individual particles. We apply a unique aerosol model that fully resolves the composition of individual particles, enabling us to evaluate how knowledge of particle-level composition impacts light absorption by BC. In particular, we compare absorption enhancement by BC using particle-resolved composition and absorption enhancement by BC assuming the mass fraction of each aerosol component is the same for all particles. Absorption enhancement (*E*_abs_) is defined as the absorption by mixed BC-containing particles relative to absorption by the same amount of BC in pure, uncoated particles. While other studies show modelled absorption depends strongly on the representation of particle morphology (for example, refs 26, 27, 28) and on absorption by organic carbon coatings (for example, refs 16, 17), our findings suggest that diversity in particle composition also plays an important role. We show that oversimplifying the representation of particle composition leads to overestimation in absorption enhancement, which is one factor affecting atmospheric heating by BC. To identify factors influencing light absorption by BC, we performed a nonparametric regression on a series of simulations to identify the key independent variables that most affect *E*_abs_. Through this nonparametric analysis, we derived a relationship for *E*_abs_ as a function of the population-averaged mass fraction of aerosol components and the environmental relative humidity (RH), such that the relationship for *E*_abs_ accounts for particle-level variation in composition but depends only on variables that many global models already track. Finally, by applying this nonparametric relationship to output from a global model, we demonstrate that light absorption by BC at the global scale depends strongly on particle-scale diversity in composition.

## Results

### Absorption by BC in individual particles

The particle-resolved model PartMC-MOSAIC^29^,^30^ was used to simulate diversity in per-particle composition for populations of BC-containing particles. We considered 100 week-long simulations of urban air parcels, varying gas and particle emissions, background particle concentrations and meteorological conditions (Methods section). Although several particle types were included in the simulations, this study focuses on the evolution of the BC-containing particles only, which originate from combustion sources. These combustion particles were emitted as a mixture of BC and primary organic aerosol (POA), where the ratio of BC to POA varied among simulations to represent differences in the composition of emitted particles between BC sources. After their emission, the combustion particles accumulated additional coating through condensation of semi-volatile gases and coagulation with other particles.

**Figure 1:**
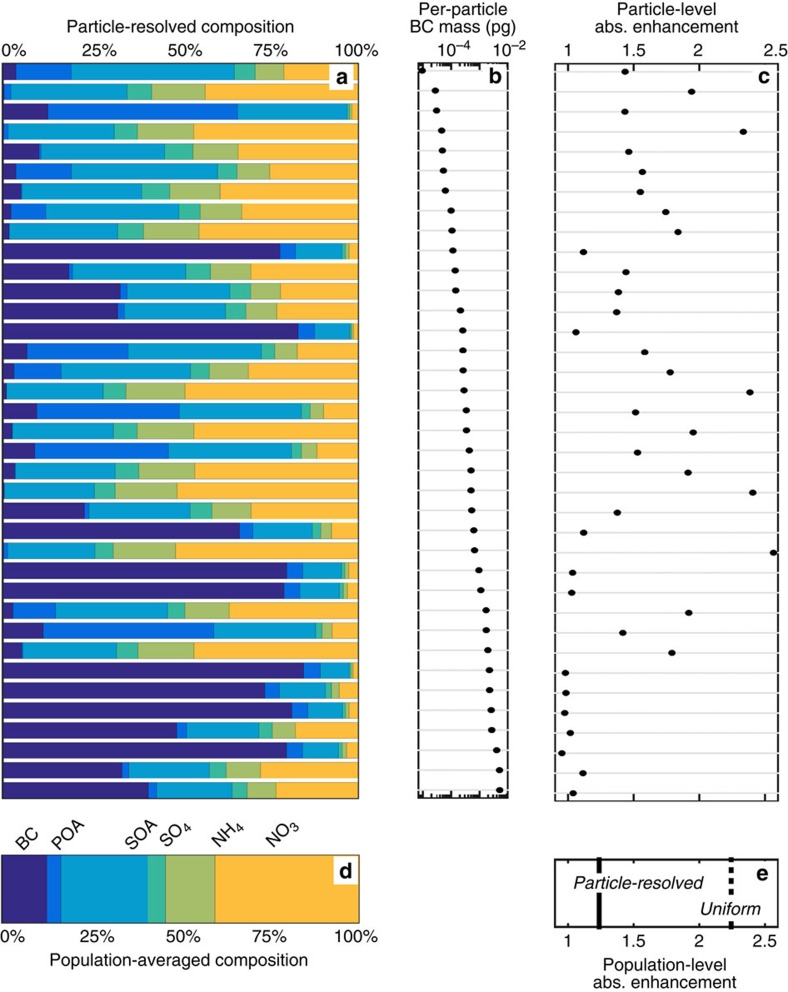
Role of particle composition on absorption enhancement in baseline population. (**a**) Per-particle volume composition for subset of particles in a single population, (**b**) mass of BC contained in each particle and (**c**) the absorption enhancement by the BC within each particle, all shown for the particle-resolved representation, as well as the (**d**) volume composition averaged across all BC-containing particles. (**e**) Overall absorption enhancement by BC in the population is computed from particle-resolved composition (solid line) and, for the same population, assuming uniform composition (dashed line).

In the particle-resolved representation of composition, each particle is different, depending on how recently it was emitted, its composition at the time it was emitted and the changes in composition that occurred since its emission. The wide variation in per-particle composition represented by the particle-resolved model is illustrated in Fig. 1a. Figure 1a shows a subset of particles sampled from a population of thousands of BC-containing particles in an aged air mass, sampled from a single scenario at 18:00 hours on the first day. Simulation settings for this scenario are outlined in Supplementary Table 1. The particle properties shown in Fig. 1a are sorted according to the mass of BC contained in each particle, which is shown in Fig. 1b. From this particle-resolved model output, absorption by each individual particle was modelled as a function of its composition, the number of BC inclusions that it contains and the environmental RH (Methods section). Rather than applying the approximation that BC exists as a single inclusion at the centre of each BC-containing particle, as in the widely-used core-shell approximation, we modelled particles using the dynamic effective medium approximation (DEMA)^31^,^32^. Under this effective medium approximation, each particle is assumed to contain one or more randomly-distributed BC inclusions that are fully encompassed in the non-absorbing material, including water, where changes in number of inclusions through coagulation events are tracked by the particle-resolved model. Water uptake by each particle was modelled as a function of particle-level composition and the environmental RH using the *κ*-Köhler model^33^ (Methods section). We focus on absorption enhancement due to non-absorbing coatings and, therefore, do not consider absorption by organic carbon. Absorption enhancement by BC within individual particles is shown for RH=50% in Fig. 1c. Within the same population, particle-level absorption enhancement ranges from ∼1 (no enhancement) to >2, depending on the particle's size and composition.

Absorption enhancement for the entire BC population is the sum over the absorption cross sections of coated particles divided by the sum of the absorption cross sections of uncoated particles. Absorption enhancement by the diverse population modelled by PartMC-MOSAIC is ∼ 1.3 at RH=50% (solid line in Fig. 1e). Global aerosol models do not fully resolve this variation in particle composition. Instead, many aerosol models track one or more separate populations of BC-containing particles but assume uniform composition across all particles within each population^21^,^22^,^34^. The average composition for the example population is shown in Fig. 1d. If the amount of BC in each particle is assumed to be the same as in the particle-resolved case but all BC-containing particles are assumed to have the average composition (Fig. 1d), we find *E*_abs_>2 (dashed line in Fig. 1e), overestimating modelled absorption by 50% relative to absorption derived from particle-resolved output. Enhancement in light absorption due to radiative interactions between particles, which may also affect heating rates^35^, is not considered.

**Figure 2:**
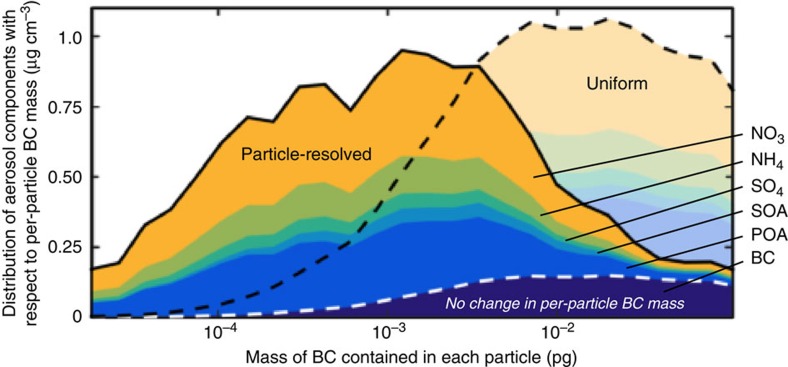
Mass distribution of aerosol components with respect to per-particle BC mass (dM_k_/d log m_BC_). With particle-resolved composition, most of the BC mass is contained in particles with large amounts of BC and small amounts of non-BC components, or thin coatings. Non-BC components are NO_3_ (orange), NH_4_ (green), SO_4_ (cyan), SOA (cerulean) and POA (blue). If uniform composition is assumed across the same BC population, the amount of non-BC material on particles containing large amounts of BC mass is increased, leading to greater BC absorption by these particles. Because these particles containing large amounts of BC dominate absorption, population-level absorption enhancement is overpredicted. The total mass of each component for each population of BC-containing particles is the same for both the uniform and particle-resolved cases.

This error in modelled absorption from the simplified representation is caused by failure to capture the diversity in composition that is simulated by the particle-resolved model. While per-particle composition varies, even for particles of the same size, the mass fraction of coating material (Fig. 1a) tends to be smaller for particles that contain large amounts of BC (Fig. 1b), corresponding to weak enhancement in light absorption by these particles (Fig. 1c). Figure 2 confirms that, across the entire diverse BC population, most of the coating material is contained in particles that have small amounts of BC mass, while most of the BC mass resides in particles with large BC inclusions and only thin coatings due to variation in coating accumulation rates as a function of particle size. For comparison, Fig. 2 also shows how the aerosol mass would be distributed if composition diversity had been neglected. Applying the constraint that all particles contain the same volume fraction of each aerosol component (uniform composition), while per-particle BC mass remains the same, causes an artificial redistribution of coating material onto particles containing large amounts of BC. This artificial increase in coating on particles containing most of the BC mass leads to overestimation of absorption by the particles that contribute most to population-level absorption. As a result, population-level absorption is greater if BC-containing particles are assumed to have identical composition than if the true diversity of the population is represented (Fig. 1e). The negative correlation between the volume fraction of dry coating with the amount of BC contained in individual particles is consistent with ambient observations^36^,^37^. This finding suggests that absorption by BC may be better represented in models that resolve variation in coatings across BC populations (for example, refs 25, 38, 39), but this potential role of mixing state on BC absorption should also be validated against observations.

### Population-level absorption enhancement

In this section, we describe a nonparametric relationship for absorption enhancement derived from a series of particle-resolved model simulations. The simulations were designed to cover a range of atmospheric conditions, varying from polluted conditions that promote rapid accumulation of coating material to conditions with low gas and particle concentrations that promote slow particle transformations (Methods section). Whereas, Figs 1 and 2 illustrated the importance of composition diversity on absorption enhancement in a single population, here we include thousands of populations, sampled at different time steps from the 100 sensitivity simulations, to determine how absorption enhancement varies with averaged properties of the particle populations. Using a nonparametric regression^40^,^41^ (Methods section), we developed a relationship for *E*_abs_ that depends on variables that many global models already track: (1) the volume fraction of dry aerosol coating that is mixed with BC-containing particles, (2) the hygroscopicity of the coating material and (3) the environmental RH. Including these three variables yielded the best prediction of *E*_abs_ in comparison with particle-resolved model results (*R*^2^=0.85). Adding other population-level variables to the regression, such as the refractive index of the coating or the mean diameter of BC inclusions, did not improve the fit.

**Figure 3:**
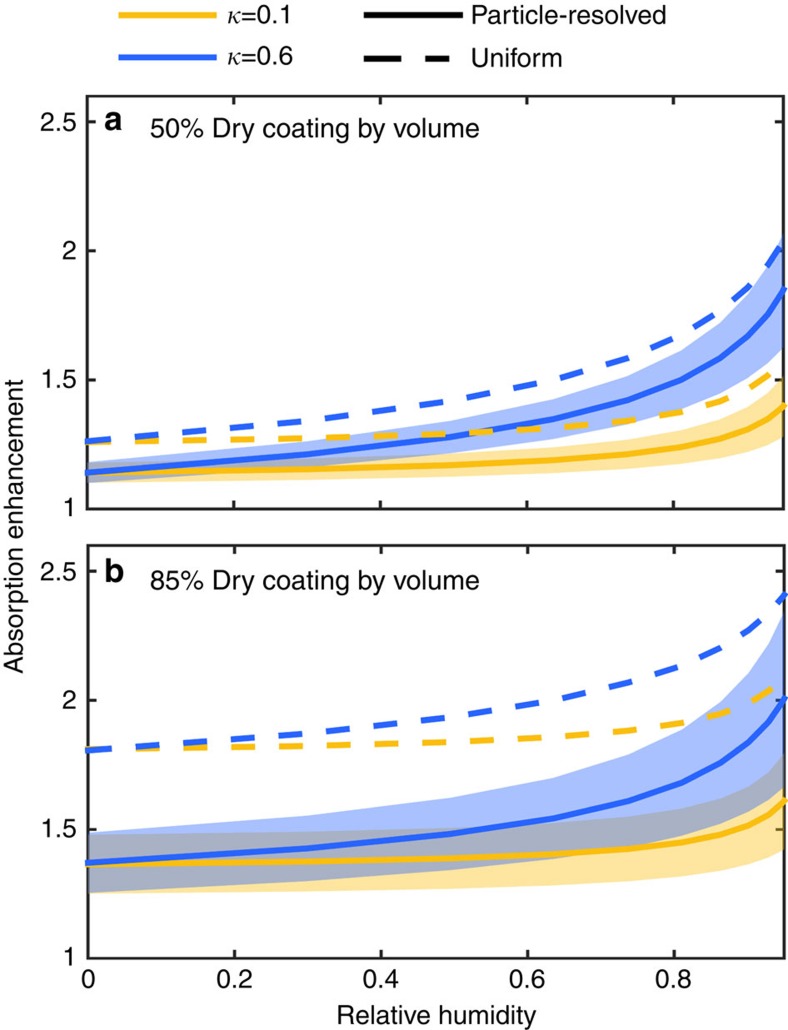
Absorption enhancement versus environmental relative humidity. Relationship between absorption enhancement (vertical axes) and environmental RH (horizontal axes) for varying average volume fraction of non-absorbing coatings (panels) and average hygroscopic parameter of the coating material (colours). Population-level absorption computed from particle-resolved composition are shown by solid lines. Shading shows the middle quartiles for the predictions, caused by differences in particle-level composition for populations that have the same population-averaged composition. Absorption enhancement on the basis of particle-resolved composition (solid lines) is compared with absorption enhancement computed from uniform mixture approximation (dashed lines).

The relationship between absorption enhancement and the independent variables is shown in Fig. 3. Absorption enhancement is shown as a function of the environmental RH (horizontal axes) for populations that contain, on average, 50% dry coating by volume (top panel) and 85% dry coating by volume (bottom panel), with coatings that are, on average, hydrophobic (*κ*_coat_=0.05, orange) or hygroscopic (*κ*_coat_=0.6, blue), representing organic and inorganic coatings, respectively. This relationship allows the prediction of absorption enhancement for any value of *f*_coat_ or *κ*_coat_, but, for simplicity, only a few combinations of *f*_coat_ and *κ*_coat_ are shown in Fig. 3.

This nonparametric relationship, which uses only population-averaged composition information, is able to reproduce the population-level absorption enhancement from the particle-resolved model with high accuracy (solid lines). Also shown in Fig. 3 is the absorption enhancement that would be predicted if BC-containing particles are assumed to have uniform composition (dashed lines). Differences between the solid and dashed lines in Fig. 3 indicate error in modelled absorption from the simplified representation of particle composition.

For thinly coated particles, such as freshly emitted particles, neglecting composition diversity yields only small error in BC absorption at all relative humidities. Comparison between the top and bottom panels of Fig. 3 demonstrates the effect of coating thickness on absorption enhancement. For the uniform composition treatment (dashed lines), absorption enhancement increases as dry coating material is added to the particles, consistent with previous modelling (for example, refs 14, 15) and laboratory measurements(for example, refs 42, 43, 44). However, when absorption is modelled at low RH using particle-resolved composition (solid lines) we find only weak increases in absorption with the amount of coating material, consistent with ambient observations^15^,^16^,^17^. Thus, the finding that a diverse particle population exhibits lower absorption than one with uniform composition holds for particle populations simulated in a wide range of relevant atmospheric conditions.

Although we find *E*_abs_<1.5 for diverse populations under dry conditions, absorption increases by as much as 50% for these populations when particles take up water at high RH. This finding suggests that absorption may be strongly enhanced in humid regions of the atmosphere, even if observations under dry conditions do not capture this enhancement. Ambient particles are dried to RH≈50% before absorption measurements, so field studies are unable to observe the response in absorption enhancement to changes in RH. The role of water uptake on absorption enhancement has been demonstrated for cloud droplets that contain BC through laboratory measurements^45^ and modelling^35^.

### Global-scale absorption enhancement

**Figure 4:**
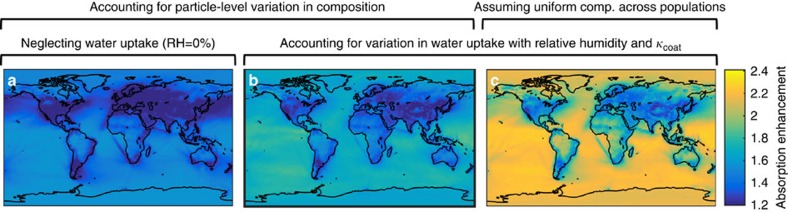
Spatial variation in absorption enhancement at the surface computed from different representations of particle composition. (**a**) On the basis of particle-resolved model data, assuming dry conditions (solid lines in Fig. 3, RH=0%), (**b**) on the basis of particle-resolved model data, accounting for water uptake (solid lines in Fig. 3, RH from global model) and (**c**) assuming uniform composition across each BC population and accounting for water uptake (dashed lines in Fig. 3, RH from global model).

The relationship for absorption enhancement shown in Fig. 3 was applied to global model fields generated by Goddard Institute for Space Studies (GISS) climate modelE^19^ coupled to Multiconfiguration Aerosol Tracker of mIXing state (MATRIX)^22^. MATRIX represents 16 aerosol modes, 7 of which contain BC (Methods section). Rather than applying the optics calculations from GISS-MATRIX, we applied the relationship shown in Figure 3 that was constructed from thousands of particle-resolved populations. Taking as inputs the RH in each location, the mass concentration of BC in each mode and the mass fraction and effective hygroscopicity parameter of dry coating for each mode from GISS-MATRIX, we used the relationship shown in Fig. 3 to estimate the global distribution in absorption enhancement by BC. From the same climate model output, we modelled (1) absorption enhancement for diverse populations but neglecting water uptake (Fig. 4a), (2) absorption enhancement for diverse populations and including water uptake (Fig. 4b) and (3) absorption enhancement for uniform composition and also including water uptake in response to RH (Fig. 4c). Comparison between Fig. 4b,c demonstrates that simplifying the representation of particle composition leads to overestimation of absorption enhancement in many locations (Fig. 4c). Using the relationship that accounts for composition diversity and for water uptake (Fig. 4b), absorption enhancement increases in response to RH, especially over the oceans.

**Figure 5:**
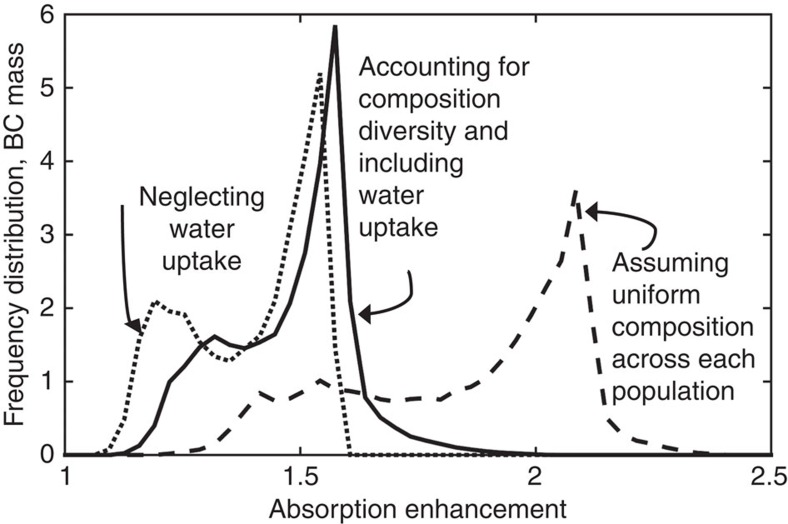
BC mass density with respect to absorption enhancement for the different representations of particle composition. Solid line shows global distribution in absorption enhancement predicted from most realistic representation, which accounts for water uptake and particle-level variation in composition. Absorption enhancement is underestimated if water uptake is neglected (dotted line) and is overestimated if we assume uniform composition across each BC population (dashed line).

Figure 5 shows the distribution in BC mass with respect to absorption enhancement level, indicating how frequently BC takes on a particular absorption enhancement level. The dotted, solid and dashed lines in Fig. 5 correspond to the treatments represented in Fig. 4a–c, respectively. Figure 5 includes BC mass throughout the entire atmosphere, whereas Fig. 4 shows *E*_abs_ by BC only at the surface.

Our best estimate of absorption enhancement, accounting for diversity and including aerosol water uptake (solid line in Fig. 5), ranges from *E*_abs_=1.1 in some continental areas to *E*_abs_ >2 in many marine areas, with a BC-weighted mean *E*_abs_ of 1.5. This overall absorption enhancement is driven, in part, by enhancement from dry aerosol coatings alone (dotted line in Fig. 5) and further enhancement due to water uptake by the dry coatings (solid line in Fig. 5). If water uptake is ignored, or if it is assumed that water uptake does not contribute to absorption enhancement, the global mean absorption enhancement is underestimated by 5%. On the other hand, if we assume uniform composition among each BC population (dashed line in Fig. 5), the global mean is overestimated of 30%.

## Discussion

Variation in particle composition within BC populations strongly affects modelled absorption. Composition diversity is not captured by aerosol schemes that assume uniform composition across BC populations, but the influence of composition diversity on absorption can be accounted for using a relationship for absorption enhancement on the basis of particle-resolved model results. If particles within a population are assumed to have uniform composition, absorption by BC under dry conditions can be enhanced by more than factor of two for BC thickly coated with non-absorbing components, consistent with previous modelling^14^,^15^. If, instead, we account for particle-level variation in composition, we find much weaker levels of absorption enhancement at low RH (*E*_abs_=1.2–1.5) and a relatively low sensitivity of *E*_abs_ to the amount of coating material, a result that is generally consistent with ambient observations^15^,^16^,^17^. Despite this limited enhancement at low RH, absorption by BC depends strongly on RH, leading to a factor of two increase in absorption at high RH compared with dry conditions. These model results suggest important roles for diversity in composition and water uptake in determining absorption enhancement that should be confirmed through observations.

## Methods

### Simulations with particle-resolved model

The particle-resolved model PartMC-MOSAIC tracks the chemical composition of thousands of individual particles as they evolve by condensation of semi-volatile substances, coagulation between particles, particle emissions and dilution with background air (Supplementary Note 1). This study presents simulations of 100 week-long parcel scenarios, which were constructed by sampling a large variable space using latin hypercube sampling^46^ (Supplementary Note 2). Although the simulations include a wide range of ambient conditions, BC size distributions and the ratio of BC to POA, which are intended to represent the range of BC sources and aging conditions, we do not specifically simulate the aerosol evolution in biomass burning plumes. The input parameters that define this variable space are provided in Supplementary Tables 1 and 2 and the resulting range in aerosol concentrations are shown in Supplementary Figs 1 and 2.

### Particle-resolved composition

For each particle, PartMC-MOSIAC tracks the mass of each constituent species *k*=1,...,*A*, including water. In this study, we extract only dry species from PartMC-MOSAIC, corresponding to *k*=1,..., *A*−1, and compute the amount water, the *A*th species, as a post-processing step. Applying the density for each species to the particle-resolved output yields the volume of each species *k* contained in each particle *i*, denoted *v*_*k*,*i*_. Each particle is then represented by a composition vector **v**_*i*_=[*v*_1,*i*_, ...*v*_*k*,*i*_, ...,*v*_*A*−1,*i*_] and the overall dry volume, *V*_d,*i*_, of each particle is given by:


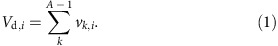


Variability in the volume fraction and hygroscopicity of coating thickness for the baseline population are shown in Supplementary Figs 3 and 4, respectively.

### Uniform mixture approximation

Under the uniform mixture approximation, each particle is represented by the vector 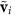. The volume of each species *k* in each particle *i* is adjusted such that the volume fraction of each dry component is the same for all particles, while the volume of BC in each particle (*v*_b,*i*_) is the same as in the particle-resolved representation:


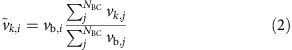


The overall dry volume under the particle-resolved representation, 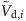, is computed from equation 1, but using the composition vector 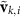 rather than **v**_*k*,*i*_. The value for 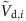 varies between particles and, for each particle, also differs from the value of *V*_d,*i*_.

### Water uptake modelled with *κ*-Köhler

The wet volume *V*_*i*_ is computed for each particle at each environmental RH using the *κ*-Köhler model^33^:


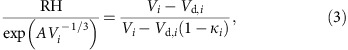


where *V*_d,*i*_ is the volume of dry aerosol components contained in particle *i* and *κ*_*i*_ is its hygroscopicity parameter. The hygroscopicity parameter *κ*_*i*_ is computed as the volume-weighted average of *κ* for each aerosol species:


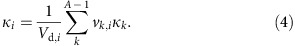


Similarly, the effective hygroscopicity corresponding to the average composition, denoted 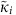, is computed from equation 4 but using the composition from the uniform mixture approximation, 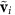. Values for *κ* for each species are provided in Supplementary Table 2. The value of *A* depends on environmental variables and properties of water, as shown in Supplementary Equation 1.

### Absorption modelled with DEMA-Mie

The absorption cross-section *σ*_abs,*i*_ of each particle *i* is computed using Mie theory, treating each BC-containing particle as an effective medium using the DEMA^31^,^32^. The Mie model takes as inputs the effective relative permittivity *ϵ*_*i*_, which is computed from DEMA, and the wet volume *V*_*i*_. The effective relative permittivity *ϵ*_*i*_ for each particle *i*=1, ..., *N*_BC_ is computed from the following non-linear equation:


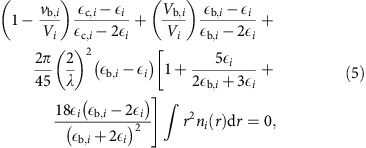


which depends on the total volume of each particle (*V*_*i*_), the volume of BC contained in each particle (*V*_b,*i*_), the relative permittivity of BC (*ϵ*_b,*i*_), the relative permittivity of each particle's non-BC coating (*ϵ*_c,*i*_) and the number and size of BC inclusions (*n*(*r*)). The relative permittivity of the coating material, *ϵ*_c,*i*_ is computed as the volume-weighted average of the non-BC components, including water. The absorption cross-section of particles under the uniform mixture approximation, 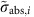, is computed in a similar manner, but using 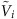 rather than *V*_*i*_ in equation 5. See Supplementary Note 3.

Enhancement in light absorption by BC within an individual particle *i* is then given by:


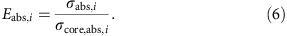


Absorption enhancement at the population-level, considering the all BC-containing particles, is computed as:


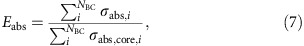


where *σ*_abs,*i*_ is the absorption coefficient of each BC-containing particle *i*=1, ..., *N*_BC_, and *σ*_abs,core,*i*_ is the absorption coefficient by the same amount of BC but as a single, uncoated core.

Similarly, population-level absorption enhancement under the uniform mixture approximation is computed from the sum over 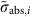 for all particles in the population:


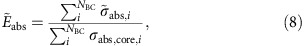


### Kernel density regression

The nonparametric relationship for *E*_abs_ shown in Fig. 3 was produced using a kernel density regression^40^,^41^ on model data from thousands of particle-resolved model populations. Populations were sampled from the 100 scenarios to represent BC-containing particles that had aged to varying degree and under a variety of conditions. The regression analysis was used to identify the set of independent variables that most affect *E*_abs_, as described in ref 47 and to construct the relationship for *E*_abs_ shown in Fig. 3 (Supplementary Note 4 and Supplementary Figure 5).

### Global climate model GISS–MATRIX

MATRIX is an aerosol scheme based on the quadrature method of moments^48^ that represents 16 aerosol modes. Operating within the general circulation climate modelE^19^, MATRIX tracks two moments of each aerosol distribution, number and mass, assuming lognormal size distributions for emissions and removal calculations. MATRIX includes sulfate, nitrate, ammonium, aerosol water, BC, POA, mineral dust and sea salt and simulates nucleation, secondary aerosol formation, condensation and coagulation. The climate simulations discussed here are identical to ref. 49. The distribution in the volume fraction of dry coating on BC, hygroscopicity of dry coating and RH are shown for surface grid cells in Supplementary Figure 6.

### Data availability

PartMC version 2.1.5 was used for the simulations in this paper, available at: http://lagrange.mechse.illinois.edu/partmc/. MOSAIC is available from Rahul Zaveri. The simulation settings are outlined in Supplementary Tables 1 and 2, and the input files are available from Laura Fierce. Code for modelling water uptake with *κ*-Köhler and absorption with DEMA are available from Laura Fierce. Model data simulated with GISS-MATRIX are available from Susanne Bauer.

## Additional information

**How to cite this article:** Fierce, L. *et al*. Black carbon absorption at the global scale is affected by particle-scale diversity in composition. *Nat. Commun.* 7:12361 doi: 10.1038/ncomms12361 (2016).

## Supplementary Material

Supplementary Information Supplementary Figures 1-6, Supplementary Tables 1-3, Supplementary Notes 1-4 and Supplementary References.
