# Supplementary material for: Black carbon absorption at the global scale is affected by particle-scale diversity in composition
Source: Nat Commun. 2016 Sep 1;7:12361. doi: 10.1038/ncomms12361 (PMC5025768; doi:10.1038/ncomms12361)
Supplement: Supplementary Information — Supplementary Figures 1-6, Supplementary Tables 1-3, Supplementary Notes 1-4 and Supplementary References. [file ncomms12361-s1.pdf]

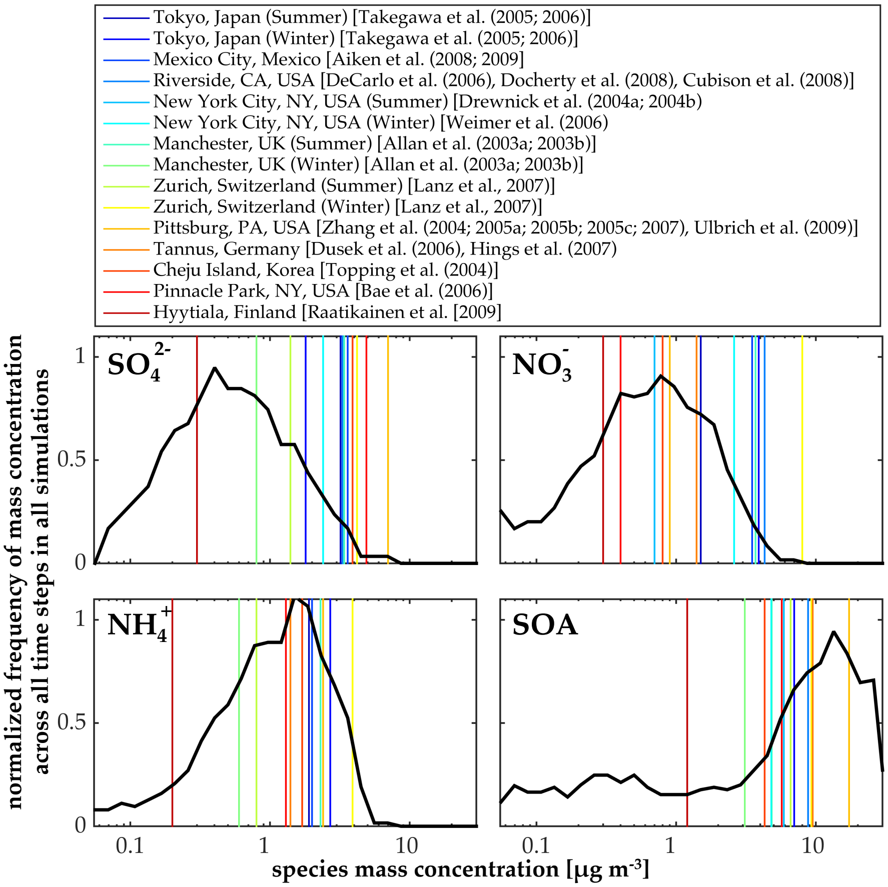

Supplementary Figure 1: Variation in mass concentration of selected aerosol species across all simulation time steps (black line), compared with observed mass concentrations from various locations (colored vertical lines). Demonstrates that the range of aerosol concentrations from simulations covers variation in observed aerosol concentrations.

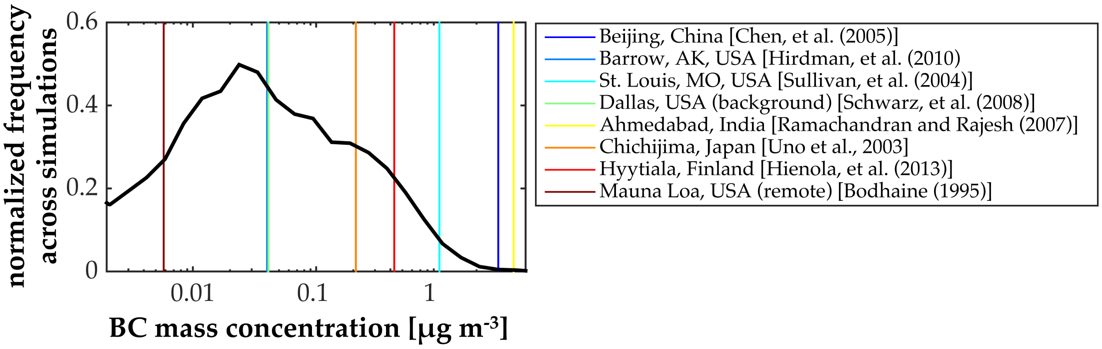

Supplementary Figure 2: Variation in black carbon mass concentration (black line), compared with observed concentrations at various locations (colored vertical lines). Demonstrates that the range of BC concentrations from simulations covers variation in observed BC concentrations.

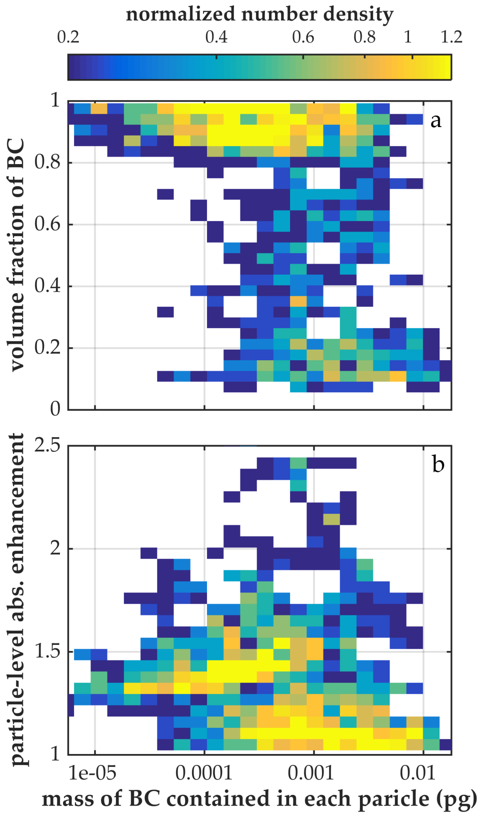

Supplementary Figure 3: Number density distribution shows how (a) per-particle volume fraction of dry coatings and (b) per-particle absorption enhancement by BC varies as a function of the mass of BC contained in each particle.

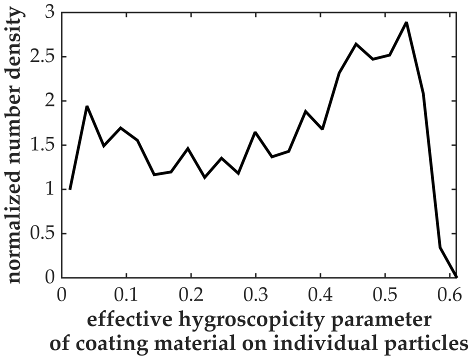

Supplementary Figure 4: Number density distribution of coating κ for baseline population shows wide variability in hygroscopic properties of BC coatings, even within a single population.

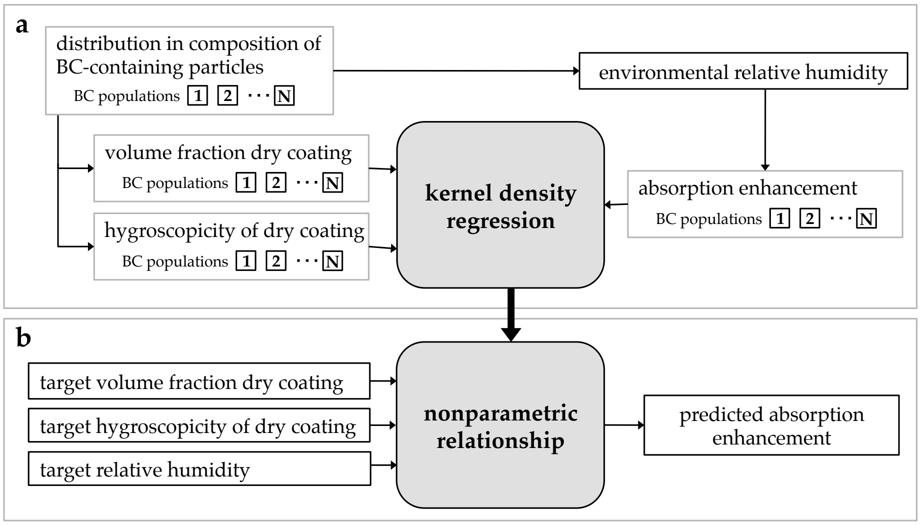

Supplementary Figure 5: Procedure for applying nonparametric regression to particle-resolved model data in order to derive a relationship for absorption enhancement as a function of population-level variables.

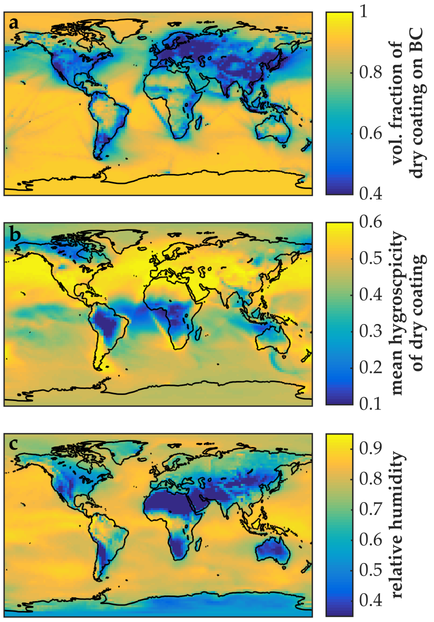

Supplementary Figure 6: Inputs to nonparametric relationship for GISS-MATRIX surface grid cells: a) mean volume fraction coating in BC modes f_{coat} and b) mean hygroscopicity parameter of coating material κ_{coat}, weighted by BC mass in each mode and c) relative humidity.

Supplementary Table 1: Properties assigned to aerosol species. Values for density and refractive index are the same as those assumed in [36]. The range in values for κ were varied dynamically to cover the range in the effective hygroscopicity parameter of coating material expected in the atmosphere, based on [38] and references therein.

aerosol species	density [kg m^{-3}]	κ	refractive index at $\lambda = 550$ nm
NO_3	1800	0.3–0.8	1.5
SO_4	1800	0.3–0.8	1.5
NH_4	1800	0.3–0.8	1.5
SOA	1400	0.001–0.5	1.45
BC	1800	0	1.82 + 0.74i
POA	1000	0.001–0.5	1.45
H_2O	1000		1.33

Supplementary Table 2: Input variables assigned in baseline scenario

**environmental variable**	**value**
temperature [K]	293
relative humidity	90%
boundary layer height [m]	230
dilution rate [s^{-1}]	1.8×10^{-5}
latitude	35°N
day of year	July 6
**aerosol characteristic**	**value**
emission rate [kg m^{-2} s^{-1}]	2.3×10^{-11}
fraction BC-free emissions	10%
fraction BC-rich emissions	65%
fraction OC-rich emissions	35%
background conc. [kg m^{-3}]	2.7×10^{-10}
fraction back. in large mode	92%
**aerosol type**	**geo. mean dia. [nm]**
BC-free	110
BC-rich	100
OC-rich	85
small background	20
large background	116
**aerosol type**	**geo. standard dev.**
BC-free	1.7
BC-rich	1.7
OC-rich	1.7
small background	1.4
large background	1.6
**aerosol type**	**mass composition**
BC-free	0% BC, 100% OC
BC-rich	97% BC, 3% OC
OC-rich	30% BC, 70% OC
background	50% OC, 35% SO_4, 15% NH_4
**emitted gas species**	**rate [mol m^{-2} s^{-1}]**
Ammonia	2×10^{-9}
Nitrogen oxide	4.5×10^{-8}
Nitrogen dioxide	2.4×10^{-9}
Sulfur dioxide	9.4×10^{-9}
Acetaldehyde	3.1×10^{-9}
Formaldehyde	3.1×10^{-9}
Ethene	3.6×10^{-8}
Terminal olefin carbons	1.2×10^{-8}
Internal olefin carbons	1.2×10^{-8}
Toluene	2×10^{-8}
Xylene	1.2×10^{-8}
Acetone	6×10^{-9}
Paraffin carbon	4.6×10^{-7}
Isoprene	1.1×10^{-9}
Methanol	1.4×10^{-9}
Alcohols	1.7×10^{-8}

Supplementary Table 3: Ranges over which input parameters are varied in sensitivity scenarios

**environmental variable**	**value**
temperature [K]	253 to 313
relative humidity	0% to 99%
boundary layer height [m]	100 to 400
dilution rate [s^{-1}]	1×10^{-5} to 2×10^{-5}
latitude range	0°N to 80°N
day of year	Jan. 1 to Dec. 31
**aerosol characteristic**	**value**
emission rate [kg m^{-2} s^{-1}]	1×10^{-12} to 3×10^{-11}
fraction of emissions BC-free	0% to 70%
of BC emissions, fraction BC-rich	0% to 100%
background conc. [kg m^{-3}]	2×10^{-10} to 5×10^{-8}
fraction back. in large mode	90% to 99%
**aerosol type**	**geo. mean dia. [nm]**
BC-free	100 to 200
BC-rich	50 to 150
OC-rich	50 to 150
**emitted gas species**	**rate [mol m^{-2} s^{-1}]**
Ammonia	0 to 2×10^{-7}
Nitrogen oxide	0 to 6.4×10^{-7}
Nitrogen dioxide	0 to 3.36×10^{-8}
Sulfur dioxide	0 to 1×10^{-7}
Acetaldehyde	0 to 5.2×10^{-10}
Formaldehyde	0 to 5.2×10^{-10}
Ethene	0 to 6×10^{-9}
Terminal olefin carbons	0 to 1.94×10^{-9}
Internal olefin carbons	0 to 1.94×10^{-9}
Toluene	0 to 3.2×10^{-9}
Xylene	0 to 1.92×10^{-9}
Acetone	0 to 1×10^{-9}
Paraffin carbon	0 to 7.6×10^{-8}
Isoprene	0 to 1.86×10^{-10}
Methanol	0 to 2.28×10^{-10}
Alcohols	0 to 2.76×10^{-9}

Supplementary Note 1: Particle-resolved model PartMC-MOSAIC simulates the evolution of trace gases and aerosol particles in a Lagrangian air parcel. In each simulation, the model tracks the mass composition of each simulated particle as particle populations evolve through emissions, dilution with background air, coagulation between particles, and gas-particle mass transfer. Emissions, dilution, and coagulation are simulated stochastically with PartMC, while gas- and particle-phase chemistry and gas-particle mass transfer are simulated deterministically with MOSAIC. The position of each particle is not tracked and, instead, the parcel is assumed to be well-mixed. MOSAIC includes modules for gas-phase photochemistry [1], particle-phase thermodynamics [2, 3], and gas-particle mass transfer [4]. Secondary organic aerosol formation is simulated in MOSAIC using the SORGAM scheme [5]. The coupled model includes all relevant aerosol species, including SO_4^{2-}, NO_3^-, NH_4^+, Na, Ca, other inorganic mass (including species such as SiO_2, metal oxides, and other unmeasured or unknown inorganic species present in aerosols), black carbon, primary organic carbon, and secondary organic carbon. Sulfate formation through aqueous-phase chemistry of cloud droplets is not included in the simulations; neglecting this process could affect the distribution in coating material between large and small particles (e.g. Figures 1a and 2 of the main text) and, thereby, the population-averaged absorption enhancement E_{abs} (Figures 1d and 3 of the main text). PartMC version 2.1.5 was used for the simulations in this study, which is available at: http://lagrange.mechse.illinois.edu/partmc/. MOSAIC is available upon request from R. A. Zaveri.

Supplementary Note 2: Scenarios simulated with PartMC-MOSAIC This study describes a series of 100 scenarios, representing a variety of particle populations that have aged to varying degree under a range to atmospheric conditions. Each simulation included approximately 5000 computational particles and was one week in duration. Although the simulation settings differed, the structure of the scenarios was the same. In all scenarios, we assumed the air parcels represent a slice of a well-mixed boundary layer during the day and a slice of the residual layer at night. All simulations started at 6:00 am, at which time the air parcel contained only background air. From 6:00 am until 6:00 pm on the first day particles and gases were emitted into the air parcel. All emissions were discontinued at 6:00 pm, at which time the parcel was assumed to enter the residual layer and was decoupled from fresh emissions, but all other processes continued until the simulations ended.

The first part of the manuscript describes a baseline population, sampled from a single simulation at 6:00 pm on the first day ($t = 12$ hours). The input parameters for this scenario are outlined in Table 2. In the sensitivity scenarios, thirty input parameters were varied, including environmental conditions, the magnitude and characteristics of aerosol emissions, the background aerosol concentration, and the emission rates of gas-phase species. The range in each of these input parameters is given in Table 3. Latin hypercube sampling [37] was used to select 100 scenarios from the infinite possible combinations. These 100 scenarios represent a range of atmospheric aging conditions, causing the evolution of particle sizes and composition by condensation and coagulation to proceed at different rates. Input files required to run all 100 scenarios are available from the authors.

SI Figure 1 shows that the 100 scenarios cover the observed variation in surface concentrations of key aerosol species. The distribution in aerosol mass concentration within these simulations is shown for selected species by the black curve in Figure 1, which includes all time steps in all 100 scenarios. Surface observations of these aerosol species are indicated by the colored vertical lines in Figure 1. Similarly, Figure 2 shows the distribution in BC mass concentrations across the simulations (black line) and the corresponding observations (colored vertical lines). Figures 1 and 2 illustrate that aerosol concentrations simulated in the sampled scenarios cover the observed variation in surface aerosol concentrations.

Supplementary Note 3: Offline modeling of particle optical properties Absorption enhancement by BC in each population was computed offline from per-particle composition data. Each particle's absorption cross section is computed using a combination of models. Computing the absorption cross section of an individual particle from the mass composition provided by PartMC-MOSAIC requires the density, hygroscopicity parameter, and refractive index for each aerosol species. The value of the hygroscopicity parameter κ is uncertain for various aerosol species, so we considered a range in the value of κ for each species. The density, range in κ, and refractive index at $\lambda = 550$ nm is given in Table 1.

To explore particle absorption across a range of relative humidity levels, we used the κ-Köhler model [38] to find the volume of water contained in each particle. The overall particle wet volume is determined through solution of Equation 3 of the main text, which depends on particle's dry volume, its effective hygroscopicity parameter, and the environmental relative humidity. The value of A given in Equation 3 of the main text depends on the temperature (T), the universal gas constant (R), the molecular weight of water M_{W}, the density of water ρ_{w}, and

the surface tension of the air-water interface ($\sigma_{\rm w}$), and is given by:

$$A = \frac{4\sigma_{\rm w} M_{\rm w}}{RT\rho_{\rm w}}. \tag{1}$$

The Dynamic Effective Medium Approximation (Equation 5 of the main text) [39, 40] is used to determine the effective relative permittivity ϵ_i of each BC-containing particle, where BC is contained in one or more randomly-distributed inclusions within an otherwise homogeneous particle. Each particle's wet size and effective permittivity varies as particles take up water with relative humidity, causing absorption to also vary with relative humidity. The absorption cross section of each particle is modeled as a function of its wet volume $V_{\rm d}$ and its effective relative permittivity ϵ_i with the Lorenz-Mie solution to Maxwell's equations. The same procedure is applied to compute absorption enhancement by each particle under the uniform composition approximation, but using the volume composition corresponding to the averaged population ($\bar{\mathbf{v}}_i$).

The particle-level and population-level absorption enhancement factors given Equations 6–8 of the main text indicate the ratio between absorption by BC in particles mixed with other components, including water, relative to absorption by uncoated BC. The absorption cross section of uncoated BC inclusions is computed using the Mie-Lorentz model, assuming the BC in each particle forms a single homogeneous sphere. As described in Equations 7 and 8 of the main text, the population-levels absorption enhancement under the particle-resolved composition and uniform composition representations, respectively, is given by the sum over the absorption cross section of coated BC relative to the sum over the absorption cross section of uncoated BC.

For the population of BC-containing particles shown in in Figures 1 and 2 of the main text, supplemental Figure 3a shows the distribution in particle number with respect to the mass of BC contained in each particle (horizontal axis) and the volume fraction of coating contained in each particle (vertical axis), whereas Figure 3b shows the distribution with respect to per-particle BC mass (horizontal axis) and per-particle absorption enhancement (vertical axis). For this same population, Figure 4 shows that the effective hygroscopicity parameter κ of BC coatings varies widely across BC-containing particles, even for particles within the same population.

Although the particle-resolved results reveal variability in coating thickness, even for particles of the same size, Figures 1 and 2 of the main text show that the mass of coating material associated with BC-containing particles tends to vary according with per-particle BC mass, such that most of the coating mass tends to be contained in particles containing small amounts of BC. This variation in the volume fraction of coating material between particles containing small and large amounts of BC is the result of two processes: condensation and coagulation. PartMC-MOSAIC simulates Brownian coagulation, such that the smallest BC-containing particles frequently coagulate with large background particles and, thereby, accumulate thick coatings, as described for the evolution of particle CCN properties in [41]. Condensation of semi-volatile substances also causes the volume fraction of coating material to be greater for particles with small mass-equivalent BC core diameter. Particles with small BC cores will tend to contain a greater volume fraction of coating material even in a limiting case in which the thickness of dry coating is the same across the particle population, an oversimplification that is not applied in this study but is discussed here only for illustration. For example, a particle with a mass-equivalent BC core diameter of 10 nm coated with a coating thickness of 20 nm corresponds to 99% coating by mass. On the other hand, a particle with BC core diameter of 200 nm with the same 20 nm coating thickness will contain 42% coating by mass.

Supplementary Note 4: Nonparametric regression to find relationship for population-level absorption enhancement We applied a kernel density regression [42, 43] on model data from PartMC-MOSAIC to find the relationship for absorption enhancement shown in Figure 3 of the main text. To develop this relationship, we first applied the regression to identify the independent variables that best explain variance in absorption enhancement through a similar procedure as the one described in [44]. We found that the nonparametric relationship defined in terms of the average volume fraction of dry coating, the hygroscopicity of that coating, and the environmental relative humidity, which is shown in Figure 3 of the main text, explains 85% of variance in population-level absorption enhancement.

The procedure for applying the kernel regression to construct the nonparametric relationship is illustrated in Figure 5a, and the procedure for applying this nonparametric relationship to global model fields is illustrated in Figure 5b. For each of N particle-resolved composition distributions simulated by PartMC-MOSAIC, we determined population-level absorption enhancement by BC using the procedure described in the previous section. The composition distribution was also used to find the average volume fraction of dry coating ($f_{\rm coat}$) on BC-containing particles and the average hygroscopicity of this coating ($\kappa_{\rm coat}$). The kernel regression combines all of this population-level data to find the expected value of absorption enhancement, $\hat{E}_{\rm abs}$, for a population of BC-containing particles given

their bulk composition and the relative humidity to which they are exposed:

$$\hat{E}_{\text{abs}} = \frac{\sum_{j=1}^{N} K_f(f_{\text{coat},j} - \hat{f}_{\text{coat}}) K_\kappa(\kappa_{\text{coat},j} - \hat{\kappa}_{\text{coat}}) K_R(R_{\text{w},i} - \hat{R}_{\text{w}}) E_{\text{abs},j}}{\sum_{j=1}^{N} K_f(f_{\text{coat},j} - \hat{f}_{\text{coat}}) K_\kappa(\kappa_{\text{coat},j} - \hat{\kappa}_{\text{coat}}) K_R(R_{\text{w},i} - \hat{R}_{\text{w}})}, \tag{2}$$

where $\hat{f}_{\text{coat}}$, $\hat{\kappa}_{\text{coat}}$, and $\hat{R}_{\text{w}}$ are the target values for f_{coat}, κ_{coat}, and R_{w} at which the regression is performed and K_f, K_κ, and K_R are the kernel functions with respect to f_{coat}, κ_{coat}, and R_{w}, respectively. Here, we apply a Gaussian kernel in each dimension. For example, the kernel function with respect to K_f is given by:

$$K_f\left(f_{\text{coat},j} - \hat{f}_{\text{coat}}\right) = \frac{1}{\sqrt{2\pi} h_f} \exp\left(- \frac{f_{\text{coat},j} - \hat{f}_{\text{coat}}}{2h_f^2} \right), \tag{3}$$

where the standard deviation h_f is the kernel bandwidth. The bandwidth h in each dimension was estimated using Silverman's rule of thumb [35], such that h depends on the number of independent variables, the standard deviation of each independent variable, and the total number of data points. Refer to [44] for further details on the application of nonparametric regression to PartMC-MOSAIC data.

The nonparametric relationship developed from the regression on many particle-resolved populations was used to estimate the value of E_{abs} using global model fields for the independent variables, given by $\hat{f}_{\text{coat}}$, $\hat{\kappa}_{\text{coat}}$, and $\hat{R}_{\text{w}}$ in Equation 2, using the procedure shown in Figure 5b. For each of 7 BC modes simulated by the global aerosol scheme GISS-MATRIX, we estimated population-level absorption enhancement E_{abs}. Values for f_{coat} and κ_{coat} for each mode in each location and R_{w} in each location were extracted directly from GISS-MATRIX. Supplementary Figure 6 shows mean values for f_{coat} and κ_{coat}, weighted by BC mass and R_{w}, for surface-level grid cells. Values for κ_{coat} are computed using the composition of each MARIX mode, assuming κ of 0.6, , and 0 for sulfate, dust, and organic aerosol, respectively. The representation of aerosol chemistry and dynamics varies between global models, leading to differences in f_{coat} and κ_{coat}, and thereby E_{abs}, depending on the modeled species and treatments of particle size distributions and mixing state.

Supplementary References

[1] Zaveri, R. and Peters, L. (1999). A new lumped structure photochemical mechanism for large-scale applications. *Journal of Geophysical Research*, 104(D23):30387–30.

[2] Zaveri, R., Easter, R., and Wexler, A. (2005a). A new method for multicomponent activity coefficients of electrolytes in aqueous atmospheric aerosols. *Journal of Geophysical Research*, 110(D2):D02201.

[3] Zaveri, R., Easter, R., and Peters, L. (2005b). A computationally efficient multicomponent equilibrium solver for aerosols (MESA). *Journal of Geophysical Research*, 110(D24):D24203.

[4] Zaveri, R., Easter, R., Fast, J., and Peters, L. (2008). Model for simulating aerosol interactions and chemistry (MOSAIC). *Journal of Geophysical Research*, 113:D13204.

[5] Schell, B., Ackermann, I. J., Hass, H., Binkowski, F. S., and Ebel, A. (2001). Modeling the formation of secondary organic aerosol within a comprehensive air quality model system. *Journal of Geophysical Research: Atmospheres*, 106:28.

[6] Sun, J., Zhang, Q., Canagaratna, M. R., Zhang, Y., Ng, N. L., Sun, Y., Jayne, J. T., Zhang, X., Zhang, X., and Worsnop, D. R. (2010). Highly time-and size-resolved characterization of submicron aerosol particles in beijing using an aerodyne aerosol mass spectrometer. *Atmospheric Environment*, 44(1):131–140.

[7] Takegawa, N., Miyazaki, Y., Kondo, Y., Komazaki, Y., Miyakawa, T., Jimenez, J., Jayne, J., Worsnop, D., Allan, J., and Weber, R. (2005). Characterization of an aerodyne aerosol mass spectrometer (ams): Intercomparison with other aerosol instruments. *Aerosol Science and Technology*, 39(8):760–770.

[8] Takegawa, N., Miyakawa, T., Kondo, Y., Jimenez, J., Zhang, Q., Worsnop, D., and Fukuda, M. (2006). Seasonal and diurnal variations of submicron organic aerosol in tokyo observed using the aerodyne aerosol mass spectrometer. *Journal of Geophysical Research: Atmospheres*, 111(D11).

[9] Aiken, A., Salcedo, D., Cubison, M. J., Huffman, J., DeCarlo, P., Ulbrich, I. M., Docherty, K. S., Sueper, D., Kimmel, J., Worsnop, D. R., et al. (2009). Mexico city aerosol analysis during milagro using high resolution aerosol mass spectrometry at the urban supersite (t0)–part 1: Fine particle composition and organic source apportionment. *Atmospheric Chemistry and Physics*, 9(17):6633–6653.

[10] DeCarlo, P., Dunlea, E., Kimmel, J., Aiken, A., Sueper, D., Crounse, J., Wennberg, P., Emmons, L., Shinozuka, Y., Clarke, A., et al. (2008). Fast airborne aerosol size and chemistry measurements above mexico city and central mexico during the milagro campaign. *Atmos. Chem. Phys*, 8(14):4027–4048.

[11] Docherty, K. S., Stone, E. A., Ulbrich, I. M., DeCarlo, P. F., Snyder, D. C., Schauer, J. J., Peltier, R. E., Weber, R. J., Murphy, S. M., Seinfeld, J. H., et al. (2008). Apportionment of primary and secondary organic aerosols in southern california during the 2005 study of organic aerosols in riverside (soar-1). *Environmental science & technology*, 42(20):7655–7662.

[12] Cubison, M., Alfarra, M., Allan, J., Bower, K., Coe, H., McFiggans, G., Whitehead, J., Williams, P., Zhang, Q., Jimenez, J., et al. (2006). The characterisation of pollution aerosol in a changing photochemical environment. *Atmospheric Chemistry and Physics*, 6(12):5573–5588.

[13] Drewnick, F., Schwab, J. J., Jayne, J. T., Canagaratna, M., Worsnop, D. R., and Demerjian, K. L. (2004a). Measurement of ambient aerosol composition during the PMTACS-NY 2001 using an aerosol mass spectrometer. part I: Mass concentrations special issue of aerosol science and technology on findings from the fine particulate matter supersites program. *Aerosol Science and Technology*, 38(S1):92–103.

[14] Drewnick, F., Jayne, J. T., Canagaratna, M., Worsnop, D. R., and Demerjian, K. L. (2004b). Measurement of ambient aerosol composition during the PMTACS-NY 2001 using an aerosol mass spectrometer. part II: Chemically speciated mass distributions special issue of aerosol science and technology on findings from the fine particulate matter supersites program. *Aerosol Science and Technology*, 38(S1):104–117.

[15] Weimer, S., Drewnick, F., Hogrefe, O., Schwab, J. J., Rhoads, K., Orsini, D., Canagaratna, M., Worsnop, D. R., and Demerjian, K. L. (2006). Size-selective nonrefractory ambient aerosol measurements during the particulate matter technology assessment and characterization study–new york 2004 winter intensive in new york city. *Journal of Geophysical Research: Atmospheres*, 111(D18).

[16] Allan, J. D., Jimenez, J. L., Williams, P. I., Alfarra, M. R., Bower, K. N., Jayne, J. T., Coe, H., and Worsnop, D. R. (2003a). Quantitative sampling using an Aerodyne aerosol mass spectrometer 1. Techniques of data interpretation and error analysis. *Journal of Geophysical Research: Atmospheres (1984–2012)*, 108(D3).

[17] Allan, J. D., Alfarra, M. R., Bower, K. N., Williams, P. I., Gallagher, M. W., Jimenez, J. L., McDonald, A. G., Nemitz, E., Canagaratna, M. R., Jayne, J. T., et al. (2003b). Quantitative sampling using an Aerodyne aerosol mass spectrometer 2. Measurements of fine particulate chemical composition in two uk cities. *Journal of Geophysical Research: Atmospheres (1984–2012)*, 108(D3).

[18] Lanz, V., Alfarra, M., Baltensperger, U., Buchmann, B., Hueglin, C., and Prévôt, A. (2007). Source apportionment of submicron organic aerosols at an urban site by factor analytical modelling of aerosol mass spectra. *Atmospheric Chemistry and Physics*, 7(6):1503–1522.

[19] Zhang, Q., Stanier, C. O., Canagaratna, M. R., Jayne, J. T., Worsnop, D. R., Pandis, S. N., and Jimenez, J. L. (2004). Insights into the chemistry of new particle formation and growth events in pittsburgh based on aerosol mass spectrometry. *Environmental science & technology*, 38(18):4797–4809.

[20] Zhang, Q., Canagaratna, M. R., Jayne, J. T., Worsnop, D. R., and Jimenez, J.-L. (2005a). Time-and size-resolved chemical composition of submicron particles in pittsburgh: Implications for aerosol sources and processes. *Journal of Geophysical Research: Atmospheres)*, 110(D7).

[21] Zhang, Q., Alfarra, M. R., Worsnop, D. R., Allan, J. D., Coe, H., Canagaratna, M. R., and Jimenez, J. L. (2005b). Deconvolution and quantification of hydrocarbon-like and oxygenated organic aerosols based on aerosol mass spectrometry. *Environmental Science & Technology*, 39(13):4938–4952.

[22] Zhang, Q., Worsnop, D., Canagaratna, M., and Jimenez, J. (2005c). Hydrocarbon-like and oxygenated organic aerosols in pittsburgh: insights into sources and processes of organic aerosols. *Atmospheric Chemistry and Physics*, 5(12):3289–3311.

[23] Zhang, Q., Jimenez, J. L., Worsnop, D. R., and Canagaratna, M. (2007). A case study of urban particle acidity and its influence on secondary organic aerosol. *Environmental science & technology*, 41(9):3213–3219.

[24] Dusek, U., Frank, G., Hildebrandt, L., Curtius, J., Schneider, J., Walter, S., Chand, D., Drewnick, F., Hings, S., Jung, D., et al. (2006). Size matters more than chemistry for cloud-nucleating ability of aerosol particles. *Science*, 312(5778):1375–1378.

[25] Hings, S. S., Walter, S., Schneider, J., Borrmann, S., and Drewnick, F. (2007). Comparison of a quadrupole and a time-of-flight aerosol mass spectrometer during the feldberg aerosol characterization experiment 2004. *Aerosol Science and Technology*, 41(7):679–691.

[26] Topping, D., Coe, H., McFiggans, G., Burgess, R., Allan, J., Alfarra, M., Bower, K., Choularton, T., Decesari, S., and Facchini, M. C. (2004). Aerosol chemical characteristics from sampling conducted on the island of Jeju, Korea during ACE Asia. *Atmospheric Environment*, 38(14):2111–2123.

[27] Bae, M.-S., Demerjian, K. L., and Schwab, J. J. (2006). Seasonal estimation of organic mass to organic carbon in pm 2.5 at rural and urban locations in new york state. *Atmospheric Environment*, 40(39):7467–7479.

[28] He, K., Yang, F., Ma, Y., Zhang, Q., Yao, X., Chan, C. K., Cadle, S., Chan, T., and Mulawa, P. (2001). The characteristics of PM 2.5 in Beijing, China. *Atmospheric Environment*, 35(29):4959–4970.

[29] Venkataraman, C., Reddy, C. K., Josson, S., and Reddy, M. S. (2002). Aerosol size and chemical characteristics at Mumbai, India, during the INDOEX-IFP (1999). *Atmospheric Environment*, 36(12):1979–1991.

[30] Sullivan, A., Weber, R., Clements, A., Turner, J., Bae, M., and Schauer, J. (2004). A method for on-line measurement of water-soluble organic carbon in ambient aerosol particles: Results from an urban site. *Geophysical Research Letters*, 31(13).

[31] Schwarz, J., Gao, R., Spackman, J., Watts, L., Thomson, D., Fahey, D., Ryerson, T., Peischl, J., Holloway, J., Trainer, M., Frost, G. J., Baynard, T., Lack, D. A., de Gouw, J. A., Warneke, C., and Del Negro, L. A. (2008). Measurement of the mixing state, mass, and optical size of individual black carbon particles in urban and biomass burning emissions. *Geophysical Research Letters*, 35(13).

[32] Putaud, J.-P., Van Dingenen, R., Baltensperger, U., Brüggemann, E., Charron, A., Facchini, M. C., Decesari, S., Fuzzi, S., Gehrig, R., Hansson, H.-C., et al. (2003). A European Aerosol Phenomenology: physical and chemical characteristics of particulate matter at kerbside, urban, rural and background sites in Europe.

[33] Hienola, A., Pietikäinen, J.-P., Jacob, D., Pozdun, R., Petäjä, T., Hyvärinen, A.-P., Sogacheva, L., Kerminen, V.-M., Kulmala, M., and Laaksonen, A. (2013). Black carbon concentration and deposition estimations in Finland by the regional aerosol-climate model REMO-HAM. *Atmospheric Chemistry and Physics*, 13(8):4033.

[34] Bodhaine, B. A. (1995). Aerosol absorption measurements at Barrow, Mauna Loa and the South Pole. *Journal of Geophysical Research: Atmospheres (1984–2012)*, 100(D5):8967–8975.

[35] Silverman, B. (1986). *Density estimation for statistics and data analysis*, volume 26. Chapman & Hall/CRC.

[36] Zaveri, R. A., Barnard, J. C., Easter, R. C., Riemer, N., and West, M. (2010). Particle-resolved simulation of aerosol size, composition, mixing state, and the associated optical and cloud condensation nuclei activation properties in an evolving urban plume. *Journal of Geophysical Research: Atmospheres*, 115(D17).

[37] McKay, M. D., Beckman, R. J., and Conover, W. J. (1979). Comparison of three methods for selecting values of input variables in the analysis of output from a computer code. *Technometrics*, 21(2):239–245.

[38] Petters, M. D. and Kreidenweis, S. M. (2007). A single parameter representation of hygroscopic growth and cloud condensation nucleus activity. *Atmospheric Chemistry and Physics*, 7(8):1961–1971.

[39] Chylek, P., Ramaswamy, V., and Cheng, R. J. (1984). Effect of graphitic carbon on the albedo of clouds. *Journal of the Atmospheric Sciences*, 41(21):3076–3084.

[40] Jacobson, M. Z. (2006). Effects of externally-through-internally-mixed soot inclusions within clouds and precipitation on global climate. *The Journal of Physical Chemistry A*, 110(21):6860–6873.

[41] Fierce, L., Riemer, N., and Bond, T. C. (2013). When is cloud condensation nuclei activity sensitive to particle characteristics at emission? *Journal of Geophysical Research*, 118(24).

[42] Nadaraya, E. A. (1964). On estimating regression. *Theory of Probability & Its Applications*, 9(1):141–142.

[43] Watson, G. S. (1964). Smooth regression analysis. *Sankhyā: The Indian Journal of Statistics, Series A*, pages 359–372.

[44] Fierce, L., Riemer, N., and Bond, T. C. (2015). Explaining variance in black carbon's aging timescale. *Atmospheric Chemistry and Physics*, 15(6):3173-3191.
